# Effect of MDMA exposure during pregnancy on cell apoptosis, astroglia, and microglia activity in rat offspring striatum

**DOI:** 10.22038/IJBMS.2022.64980.14308

**Published:** 2022-09

**Authors:** Zahra Nazari, Khadijeh Bahrehbar, Mohammad Jafar Golalipour

**Affiliations:** 1 Department of Biology, Faculty of Basic Sciences, Golestan University, Gorgan, Iran; 2 Department of Biology, Faculty of Basic Sciences, Yasuj University, Yasuj, Iran; 3 Gorgan Congenital Malformations Research Center, Golestan University of Medical Sciences, Gorgan, Iran

**Keywords:** Astrocyte, Cell death, Corpus striatum, MDMA, Microglia

## Abstract

**Objective(s):**

Ecstasy is a popular recreational psychostimulant with side effects on the central nervous system. This study examined the corpus striatum tissue of adult rats that received ecstasy during the embryonic period for histological and molecular studies.

**Materials and Methods:**

Rats were divided into control and ecstasy groups. The ecstasy group was given MDMA 15 mg/kg intraperitoneally twice daily at 8-hour intervals on days 7–15 of gestation. At the age of 15 weeks, adult offspring of both groups were examined for learning and memory study by the Morris water maze test. Then, ventral striatum tissue was harvested for TUNEL assay, Nissl staining, and real-time PCR for the expression of the *GFAP* and *CD11b*.

**Results:**

Ecstasy up-regulated the *GFAP* and *CD11b* expression in the striatum of offspring (*P˂0.05). Furthermore, the Morris water maze test showed that exposure to ecstasy significantly impaired learning and spatial memory (*P˂0.05). TUNEL assay results did not show any significant change in the number of apoptotic cells in the striatum tissue of ecstasy offspring compared with controls, while Nissl staining showed a significant decrease in the number of neurons in the ecstasy group (*P˂0.05).

**Conclusion:**

Exposure to ecstasy during pregnancy causes long-lasting changes in brain regions underlying learning and memory, including the striatum, and impaired working memory in the offspring. In addition, these data provide the first evidence that exposure to ecstasy during the embryonic period causes a persistent change in the activity of microglial cells and the number of astrocyte cells in the striatum.

## Introduction

MDMA (3,4-Methylenedioxymethamphetamine), commonly known as ecstasy, is one of the most abused drugs among young people worldwide (1, 2). The effects of ecstasy use include hallucinations, sleeplessness, emotional empathy, increased energy, high blood pressure, liver problems, panic attacks, jaundice, memory deficits, and attention deficits (3-5). Memory and learning impairment by ecstasy also results from neuronal damage and dysfunction of the nervous system (6). Ecstasy administration was reported previously to cause excessive release of neurotransmitters, such as dopamine (DA), glutamate, serotonin (5-HT), and norepinephrine in the brain, which damages the neurons (7, 8). Ecstasy can cause damage to the central nervous system (CNS), which increases the loss of neurons and alters brain functions (9). Several studies demonstrated that ecstasy also affects memory and learning by altering dopaminergic, serotonergic, and GABAergic neurons in experimental animals (10, 11). In rats and primates, administrations of ecstasy reduce serotonin levels in several brain regions, including the hippocampus, hypothalamus, striatum, and neocortex (12-14). It was reported that ecstasy is toxic for cortical neurons *in vitro* (10). A previous study demonstrated that in mouse models, continuous ecstasy use reduces dopamine and the number of dopaminergic neurons in the striatum (15). Several studies showed that ecstasy consumption leads to increased hypertrophy of microglia and astrocytes in different parts of the CNS, such as the hippocampus, striatum, and frontal cortex (16, 17). Inflammation within the brain, also known as neuroinflammation, is a response of the central nervous system (CNS) to noxious stimuli such as toxic metabolites, trauma, injury, autoimmunity, ischemia, microbial infections, neoplasms, infections, etc. (18). Neuroinflammation involves CNS glial cells such as microglia and astrocytes that can lead to neurodegenerative diseases (19). Studies indicated that methamphetamines could affect microglia and astrocytes and lead to neuroinflammation (20). 

Due to the prevalence of psychotropic drugs and the possible risks of using such drugs during pregnancy, the effects of ecstasy consumption by the mother during pregnancy on the activity of astrocytes, microglia, and cell death in neonatal striatum tissue have not been studied. Therefore, in this study, we investigate ecstasy’s effects on the striatum’s development. First, the Morris water maze examined spatial memory in adult male rats that received ecstasy from the mother’s blood during the embryonic period. Then, the activity of the microglial cells was examined by the microglial cell marker (CD11b) and the number of astrocytes by the astrocyte cell marker (GFAP). In addition, the apoptosis of stratum tissue was assessed using the TUNEL assay.

## Materials and Methods

***Experimental animals***

This experimental study was performed on 12 adult female rats. The rats were housed under a 12-12 hr light-dark cycle and had free access to food and water. The rats were randomly divided into two groups: group1, control receiving saline, and group2, receiving ecstasy. Various studies reported that MDMA neurotoxic doses in animals generally range from 5 to 40 mg/kg (9, 21, 22). In this study, the dose of 15 mg/kg MDMA (M6403, Sigma-Aldrich) was chosen based on previous studies. 

Both groups were mated with male animals and determined to have vaginal plaque on day 0 of pregnancy. Fifteen mg/kg ecstasy was solved in 0.2 ml saline (as MDMA solvent) and was injected by a 1-ml insulin syringe intraperitoneally into the ecstasy group twice daily at eight-hour intervals. The ecstasy group was exposed to DMEM from the 7th to the 15th day of gestation by dosage described above, while control rats were treated with vehicle (saline).

The control group was similarly injected with normal saline (23). The ethical committee of the Golestan University of Medical Sciences (Golestan, Iran) approved all animal experiments (ethical code: IR.goums.REC.1395.30).

***Morris water maze (MWM) test***

Adult offspring of control and ecstasy groups at the age of 15 weeks were examined for learning and memory study by MWM test. MWM evaluates the spatial learning and memory of animals (24). The Morris water maze consisted of a large circular black pool of 60 cm in height and 160 cm in diameter, filled to a depth of 30 cm with water at 24±2 °C and placed in a darkened room. 

The pool was divided into four quadrants: northeast, northwest, southeast, and southwest. The platform was kept submerged 1 cm below the water surface during the evaluation period. All animals received four training sessions with the hidden platform for five days, with 60 sec intersession intervals. Each session started by placing the rat at one of three start points while facing the pool wall. The start location was changed in each training session. The training session was finished when the animal entered the platform. The rat was replaced on the platform in 15 sec if it could not find the platform within 60 sec. During the acquisition of the spatial navigation task, both groups performed one session of four trials per day (days 1–5; trials 1–20). A probe test was performed on the sixth day (trial 21) to measure spatial memory. The platform was removed during the probe test, and animals were allowed to swim for the 60 sec. The path of the animals was monitored using a computerized video tracking system. Parameters including the time taken to reach the platform (latency) and swimming path length (SPL) in the training trials were measured. During the probe test, the percentage run time within the quadrant of the water bath where the platform had been previously located in the training trials was measured. 

***Gene expression analysis***

Real-time PCR was performed for GFAP and CD11b gene levels in the stratum tissue of both groups. For this aim, the brain tissues were obtained from the anesthetized offspring rats. As the ventral striatum plays key roles in learning via connections with the hippocampus, amygdala, and prefrontal cortex, the ventral striatum (NAc core and NAc shell) from each rat brain was dissected. 

Total RNA was isolated and purified from the ventral striatum with a total RNA purification kit (Invitrogen), followed by cDNA synthesis with a cDNA synthesis kit (Fermentas). Real-time PCR reactions were performed using SYBR Green Master Mix (Applied Biosystems) and a real-time PCR system (Corbett Life Science; Rotor-Gene 6000 instrument). We performed qRT-PCR according to previously published protocols (25). The samples were collected from three independent biological replicates. Table 1 lists the primer sequences used for qRT-PCR. 

***Apoptosis detection by the TUNEL assay***

*In situ* cell death detection kit (Roche) was used to detect apoptosis in the rat striatum according to the manufacturer’s instructions. We performed terminal deoxynucleotidyl transferase dUTP nick end labeling (TUNEL) assay according to our previously published protocols (26). The samples were examined under a light microscope following staining, and the apoptotic cells indicated green fluorescence. Rats were anesthetized and perfused with 4% paraformaldehyde in phosphate buffer (pH 7.4). Six series of coronal sections (50 µm thick) from control and MDMA rats were prepared and visualized at different levels. Average apoptotic cells from the ventral striatum were measured in the area of rostral, middle, and caudal sections.  

***Nissl staining***

Coronal sections from control and MDMA rats were rinsed in phosphate-buffered saline and then stained in 0.02% thionin acetate salt solution for 20 min. The sections were dehydrated, coverslipped, and visualized under a light microscope (Olympus U-TV0.5XC-3, Japan). Measurements were performed on every sixth Nissl-stained coronal section, extending from the ventral striatum’s rostral, middle, and caudal parts (6 histological sections per brain).

***Statistical analysis***

All experiments were conducted in at least three independent repeats. All data are shown as mean ± standard error of the mean. One-way analysis of variance was used to determine significant differences among groups with Tukey’s *post hoc* test. Mean data in each group were compared using a t-test. *P*<0.05 was considered significant.

## Results

**Figure 1:**
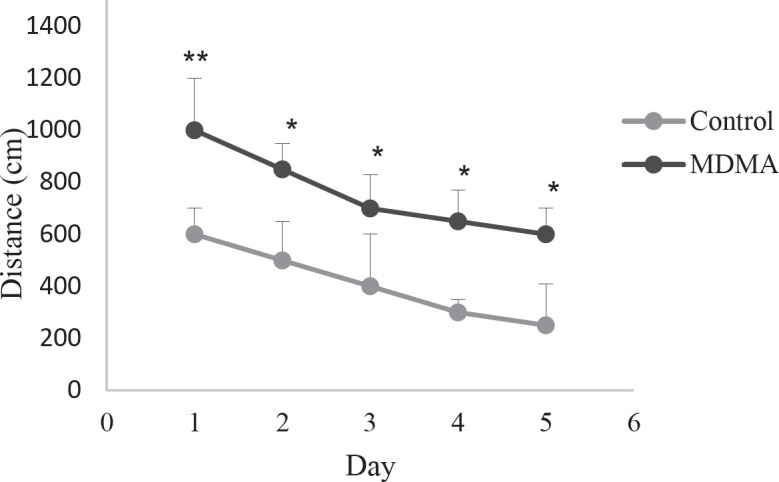
Comparison of distance traveled to the platform in the MDMA (ecstasy) group and controls on different test days. The distance traveled in the ecstasy group shows a significant increase, which indicates a decrease in learning in this group (** *P*<0.01, *P<0.05, n=6, mean± SEM)

**Figure 2:**
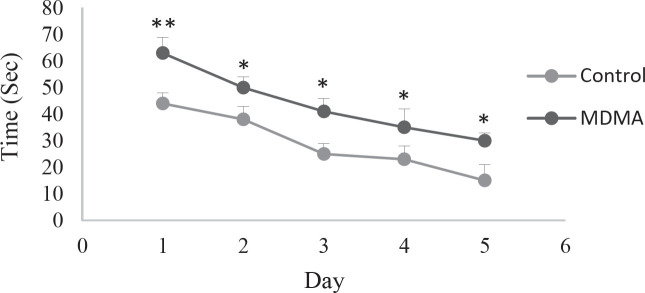
Comparison of the time elapsed to reach the platform in the control group and ecstasy on different test days. Time spent in the ecstasy group shows a significant increase, which indicates a decrease in learning in rats of this group (**P<0.01, * *P*<0.05, n=6, mean± SEM)

To simplify and better understand the behavior of animals in the experimental groups, the average distance traveled and the time spent by the study groups to reach the platform each day is shown as a point. The distance traveled during the five days of the experiment to find the platform in rats of the ecstasy group showed a significant increase compared with the control group (*P*<0.05) (Figure 1). Also, the time spent to reach the platform in rats of the ecstasy group compared with the controls showed a significant increase (* *P*<0.05) (Figure 2). In addition, the results of the six-day experiment showed that the time spent in the target quarter (a quarter where the platform was in the previous days: Q1) is less in the ecstasy group, which indicates lower spatial memory in this group.

In this study, using the real-time PCR technique, the expression of *GFAP* and *CD11b* genes in striatal tissue were examined as markers of astrocyte and microglial cells, respectively. Both genes showed increased expression in the ecstasy group, which was significant for both genes (* *P*<0.05) (Figure 4). In this experiment, the *GAPDH* gene was used as the internal control. Real-time PCR reaction was performed separately for *GFAP* and *CD11b* genes. At the end of the cycles, melting curve analysis was used to show the purity of PCR products. Figure 4 shows the relative expression levels of *GFAP* and *CD11b* mRNA between offspring of the ecstasy and control groups as a fold change.

The results of the TUNEL assay in both groups showed that the number of apoptotic cells in the striatum tissue of the offspring of the group whose mothers used ecstasy during pregnancy was not significantly changed compared with the control group (Figure 5).

We also used Nissl staining to count the total number of cells in the ventral striatum in the offspring of control and MDMA rats (Figure 6A, B), which revealed the reduced number of cells in the striatum of the MDMA group. Quantification of the nerve cells verified that MDMA exposure during pregnancy significantly reduced the number of striatal cells in the offspring (*P<0.05) (Figure 6C).

## Discussion

Although ecstasy is associated with positive feelings, it also has many side effects, such as learning and memory loss (5). The toxic effects of ecstasy that have been more exhaustively characterized in experimental animals are its neuroinflammation and neurotoxic effects (27).

Neuroinflammation involves several physiological processes that occur by microglial and astrocytic cells. These cells are critical in brain inflammation and inflammatory neurodegenerative disease (28). Microglial and astrocytic cells activate in response to a pathogenic stimulus such as tissue damage, abnormal stimulation, neurotoxins, infection, or injury. These cells secrete various proinflammatory cytokines such as IL-1, IL-6, IL-18, and TNF-α, leading to neuroinflammation (29). Abnormal microglial activation leads to pathology of several neurodegenerative diseases, including Alzheimer’s disease, Parkinson’s disease, multiple sclerosis, psychiatric disorders such as stress, depression, schizophrenia, and decreased cognitive function (30, 31). 

GFAP is an intermediate filament protein expressed in numerous types of glial cells, especially astrocytes (32, 33). Aguirre *et al*. reported that ecstasy increased GFAP in all hippocampus areas (34). The cluster of CD11b is the surface marker located on the plasma membrane of microglia. Expressions of GFAP and CD11b are used as a marker to identify glial cells and microglia, respectively. Previous studies showed that the administration of MDMA can result in microglial activation and neuroinflammation (15). Due to inflammation, excessive activation of microglial and astrocytic cells has increased the expression of GFAP and CD11b genes, respectively (35). 

 Therefore, in the present study, we assessed whether MDMA administration during pregnancy paradigm activates microglia and astrocytes in the offspring striatum. Also, we investigated the effects of MDMA on the microglial activation-induced apoptosis in offspring rats. Morris water maze task, TUNEL assay, and real-time PCR for GFAP and CD11b were performed for this aim.

As expected, administration of MDMA strongly increases the expression of CD11b genes throughout the ventral striatum of offspring of rats that were exposed to ecstasy in the pregnancy period. Increased CD11b expression indicates an increase in the activity of microglia cells and the presence of active microglia. Furthermore, our results showed that ecstasy-increased GFAP levels indicate astrocyte hypertrophy, which occurs when inflammation and degeneration occur in the nerve tissue. 

Considering that activated glial cells exert a neurotoxic effect (36) and increased microglial and astroglia reactivity induces cellular apoptosis (37), our group estimates cell apoptosis of the striatum by a TUNEL test. Although the number of apoptotic cells was higher in most ecstasy samples compared with controls, the difference was not significant overall. However, our data from Nissl staining showed that the number of neuron cells decreased in the MDMA group compared with controls (*P<0.05). Based on some reports alleging that MDMA toxicity can cause necrosis in brain cells, in this study, neuronal damage and reduced neuronal cells in the MDMA group may have occurred due to necrosis (38, 39). 

Based on the fact that neuroinflammation leads to cognitive disorders, we investigated the behavioral disorder with a Morris water maze test. Vorhees *et al*. showed that administration of ecstasy impaired spatial memory impairment in rat neonates (40). In line with previous studies (24, 40), our results of the Morris water maze test showed that the time elapsed to reach the maze platform and the distance traveled in the adult offspring of the ecstasy group to the maze platform was longer, which indicates a decrease in memory in this group. 

**Table 1:** List of primer sequences used for Real-time PCR analysis

Gene	Forward primer (5'- 3')	Reverse primer (5'- 3')	Product size	Accession number
GFAP	GCGAAGAAAACCGCATCA CC	TCTGGTGAGCCTGTATTG GGA	150	NM_017009.2
CD11b	CTGCCTCAGGGATCCGTAAAG	CCTCTGCCTCAGGAATGACATC	150	NM_012711
GAPDH	GGTGAAGGTCGGTGTGAACG	CTCGCTCCTGGAAGATGGTG	233	NM_017008.4

Gfap: Glial fibrillary acidic protein; CD11b: Cluster of differentiation molecule 11b; Gapdh: Glyceraldehyde-3-phosphate dehydrogenase

MDMA: (3,4-Methylenedioxymethamphetamine)

**Figure 3:**
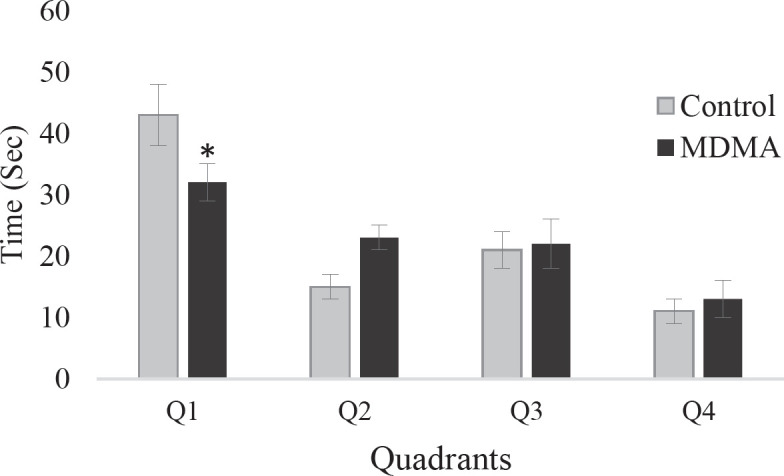
Time spent in the quarter of the platform in the control group and ecstasy on the sixth day. Time spent in the target quarter (Q1) is lower in the ecstasy group, indicating lower spatial memory in this group (* *P*<0.05). The data were obtained from 6 independent experiments and represented as mean± SEM

**Figure 4:**
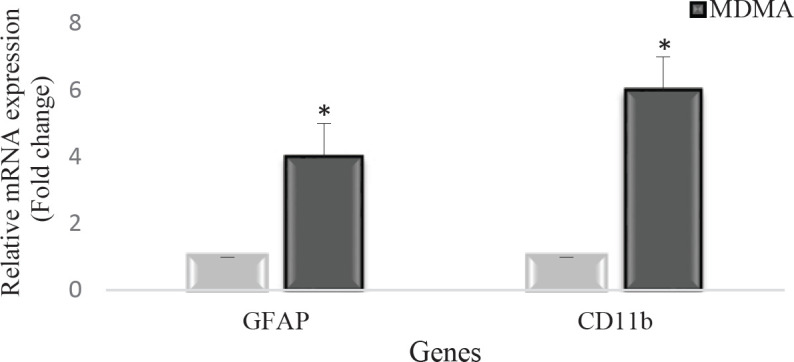
Real-time PCR results of GFAP and CD11b genes in the striatum tissue of the control and ecstasy groups, which is estimated as fold change. Both genes showed significantly increased expression in the ecstasy group

(n=6, * *P*<0.05)

**Figure 5:**
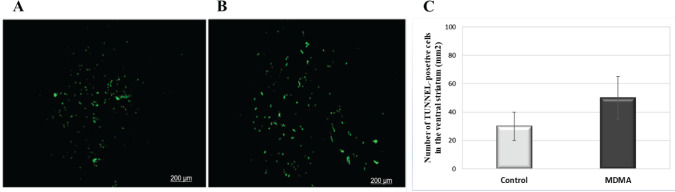
TUNEL staining of ventral striatum tissue of adult offspring rats in the control group (A) and ecstasy group (B). The number of TUNEL-positive cells in each group. Administration of ecstasy in pregnant rats did not cause a significant increase in cell apoptosis in offspring striatum tissue (C). Green color indicates apoptotic cells. The data were obtained from 6 independent experiments and represented as mean± SEM

**Figure 6:**
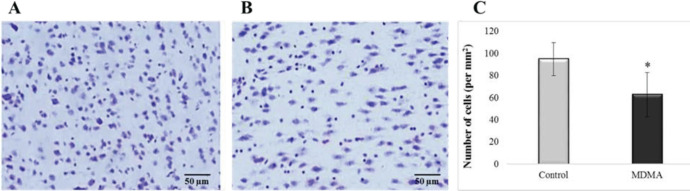
A coronal Nissl-stained ventral striatum section 50 µm thick of control and MDMA offspring rats. (A) Control group. (B) MDMA group. (C) Counts of Nissl-stained striatal neurons in each group (n = 6 rats per group). Nissl staining shows the reduced number of cells in the ventral striatum of offspring of MDMA rats. Data are expressed as the number of neurons per mm2 and presented as mean ± SEM. At least 6 images in the striatum of each rat were counted. * *P*<0.05 (unpaired two-tailed Student’s t-test)

## Conclusion

The present study results showed that the use of ecstasy during pregnancy could lead to a decrease in the number of neurons in the striatal tissue in the offspring which may occur due to increased GFAP and CD11b expression. Due to the increasing use of methamphetamine in different communities, and according to the results of the present study, it seems that the use of amphetamines during pregnancy should be avoided.

## Authors’ Contributions

MJG Supervised, directed, and managed the study. ZN Designed the experiments, performed experiments, and collected data. KB Discussed the results and strategy.

## Conflicts of Interest

The authors declare no conflict of interest.
